# The Medico-Legal, Legal, and Moral Aspects of Criminal Abortion and Infanticide

**Published:** 1894-11

**Authors:** Robert Campbell Eve

**Affiliations:** Augusta, Ga., Assistant to the Professor of Obstetrics in the Medical Department, University of Georgia; Chairman of the Section on Medical Jurisprudence, Augusta Academy of Medicine; Member of the Georgia Medical Association


### *ORIGINAL COMMUNICATIONS.*

---

### THE MEDICO-LEGAL, LEGAL, AND MORAL ASPECTS OF CRIMINAL ABORTION AND INFANTICIDE.

By ROBERT CAMPBELL EVE, M.D., Augusta, Ga.,

Assistant to the Professor of Obstetrics in the Medical Department, University of Georgia; Chairman of the Section on Medical Jurisprudence, Augusta Academy of Medicine; Member of the Georgia Medical Association.

In entering upon the task imposed by our Association, allow me to preface my remarks, by stating that there are many facts and circumstances that I can touch upon but lightly, only dealing with them in the most superficial manner.

I shall confine myself more especially to the realm of medical jurisprudence, endeavoring to emphasize, in as brief a manner as possible, the salient points involving the testimony of the medical jurist. In the first place, what is abortion?

Abortion, although spoken of in a restricted sense by most authorities, when alluding to the abnormal termination of gestation, implies the expulsion from the uterus of the products of conception prior to the viability of the fetus. Different authorities or writers have formulated such classifications as appear to them best adapted to the disturbances of pregnancy at different periods, but the one of most universal acceptance may be described as follows:

Abortion from the first to the third month, miscarriage from the third to the seventh month, and premature labor from the seventh to the ninth month. Were I writing a treatise upon abortion from a medical standpoint alone, I could but recognize that this system has its advantages, and conserves a manifold good purpose, and especially so when outlining a treatment applicable to the time, as it may be of a threatened mishap. Yet it is purely an arbitrary one, and does not concern us medico-legally; therefore, in this instance, *I shall treat the subject as defined by legal authorities, so as to make abortion encompass any interruption of pregnancy from the very*

*first moment of the impregnation of the ovule, or conception, to the full maturation of the fetus at term.*

The second question confronting us (and of vital import to the medical jurist) is, what is meant by the terms criminal abortion and infanticide?

I here quote from the law dictionary compiled by the eminent law lexicographer, Bouvier, who not alone defines criminal abortion and infanticide, but reviews the English law upon the subject. He says that abortion is the expulsion of the fetus at a period of utero-gestation, so early that it has not acquired the power of sustaining an independent life. In his résumé of the law, he states that, at common law, an attempt to destroy a child *en ventre sa mere* appears to have been held in England a misdemeanor. "In this country it has been held that it is not an indictable offence at common law to administer a drug or perform an operation upon a pregnant woman with her consent, with the intention and for the purpose of causing an abortion and premature birth of the fetus of which she is pregnant, by means of which, in fact, an abortion is caused, without averring, and proving that, at the time of administration of such drug, or performance of such operation, such woman was quick with child."

"The former English statutes on this subject distinguished between the case where the woman was quick, and was not quick, with child, and under both acts the woman must have been pregnant at the time. The terms of the recent act are, intent to procure the miscarriage of any woman, omitting the words 'being then quick with child,' etc., and it is immaterial whether the woman is or is not pregnant, if a person believes her to be, administers a drug, or uses an instrument, with the intent of producing an abortion. When, in consequence of the means used to secure an abortion, the death of the woman ensues, the crime is murder; and if a person intending to secure an abortion, does an act which causes the child to be born so much earlier than the natural time that it is born in a state much less capable of living, and afterwards dies, in consequence of its exposure to the external world, the person who by this misconduct so brings a child into the world and puts it thereby in a situation in which it cannot live, is guilty of murder, and the mere existence of the possibility that something might have been done to prevent the death does not render it less murder."

"Infanticide is the murder of a new-born infant. It is thus distinguishable from abortion and feticide, which are limited to the destruction of the life of the fetus in utero. *The crime of infanticide can be committed only after the child is wholly born.*"

Now having reviewed this phase of the subject and obtained a definition of criminal abortion and infanticide as required by law, we next proceed to the establishing, in any given case, the facts bearing upon, and the differentiation between, criminal abortion, natural abortion, or innocent abortion, authorized and justifiable abortion, and also to the establishment of infanticide. We are here confronted by a series of barriers, in the shape of important questions and considerations, which, though surmountable, claim a conspicuous rôle, and call into requisition on the part of the medical jurist indefatigable diligence and research in the scrupulously close analysis of every constituent element, for in this manner only are we enabled to render the problem soluble. I shall aim to dwell upon them in their regular order of succession, and in doing so it shall be my main object to develop the situation in its relation to what is of greatest moment to us, the law as it obtains in our own State, those laws embodied in the Code of Georgia.

What is meant by natural or innocent and authorized or justifiable abortion, and what are the facts holding a causal relation to their production, and what are the distinguishable feature deserving attention when a question of criminality arises?

Natural abortion, in law, is the expulsion of the fetus at any stage of gestation, viable or non-viable, incident to maternal dyscrasia, or disease on the one hand, or as the resultant of fetal disease on the other. As relates to the natural causes to be sought in the mother, they may be divided into constitutional and local.

Of the constitutional causes to be found: First, a toxic state of the maternal blood, either incorporating the poison in itself, as in certain fevers, lead or copper poison, etc., or the morbid products, as in jaundice, albuminuria, etc.

Second, the mother's blood may be degraded, or impoverished. Under this head may be cited anemia, over-lactation, etc.

Third, derangements of the nervous system attendant upon neurotic affections, such as chorea, extreme enervation as a consequence of obstinate, intractable vomiting, mental shock, etc.

Fourth, disturbances of the circulation attributable to mechanical causes,—*e. g.*, diseases of the liver, heart, and lungs, etc. And I should have here mentioned climacteric abortion.

Of the local causes to be found: Uterine disease, mechanical anomalies, fibroid tumors, polypi, retroversion, pressure of tumors external to uterus, etc.

The fetal diseases may be traceable to a diversity of causes: *e. g.*, diseases of the membranes of the ovum, as fatty degeneration, hydatiform degeneration, congestion, inflammation, apoplexy, and fibrous deposits; or to the embryo itself, as inflammation of serous membranes, diseases of the nervous system, kidney, liver, etc.

Here also are to be found mechanical causes, as malformation, torsion of the cord or funis, etc.

Having familiarized ourselves with the causes of natural abortion, it next rests upon us to determine whether the facts and circumstances surrounding any given case point to a fault of nature, or the intervention of crime.

From what I am able to gather from the literature to which I have had access, I am inclined to think it highly probable that the work of an expert abortionist, in the earlier months of pregnancy, might so closely simulate natural abortion as to involve quite a nice distinction—in fact, to positively preclude a positive opinion, unless corroborated by circumstantial evidence. Therefore, before rendering an ultimatum, I maintain that it is incumbent upon us, in all cases, to elicit a careful history, dating from puberty, or the first menstrual period, up to the time of, and including, the present mishap; noting carefully the character of the menses, habit as to regularity, and, in fact, every detail calculated to shed light upon the investigation.

This, conjointly with the moral environment, temperament, predisposition, and facts of a like nature, is worthy of your most careful study, and though being entitled only to collateral weight, and rendering at best but subjective evidence, still should never escape your observation, as they form a significant chapter in the antecedent history, and materially aid in unraveling the Gordian knot.

The application of the term innocent abortion includes all abortions superinduced by causes of an accidental nature, and call for no further elaboration in this paper.

The terms authorized, or justifiable, abortion are interchangeable, and are to compass all conditions necessitating the induction of abortion, where the object to be attained is the preservation of the life of the mother or child, or both.

Tidy, when reverting to the law in reference to justifiable abortion, says: "Our laws do not formally recognize the induction of premature labor by the medical practitioner. Judges, however, have always held that medical men are morally justified in inducing premature labor, provided the object be to save the life of the mother, or child, or both, as the law regards operations as justifiable, although, as Dr. Taylor remarks, no such exceptions are made in the statutes on wounding."

"The first general medical agreement upon this question appears to have been about 1756, in which Dr. Kelly informed Dr. Deneman that there was a consultation of the most eminent men at that time in London, to consider the moral rectitude of, and the advantages which might be expected from, this practice, which met with their general approbation."

The induction of abortion is justifiable, and it is recommended in the following cases: extreme narrowness of the pelvic brim, where, owing to deformity, neither the forceps nor version will succeed in bringing into the world a living child at full term; obstinate vomiting, where death seems imminent, and expedients have proven ineffectual; in some cases of pregnancy complicated with insanity; and in certain diseases of the uterus, such as cancer, etc., placenta prævia, rupture of the uterus, narrowing of the soft passages, cicatrices of the vagina, etc.

Though we are justifiable in producing abortion, should any of the above conditions, or conditions equally urgent, present themselves demanding an interference of pregnancy for the preservation of the life of the mother or child, or both, still at all times we should exert all prudence and caution, and this should be an act of conservatism born of the most mature deliberation and thought. Under no circumstances, when practicable to do otherwise, are we warranted in relying upon our own judgment, but to the contrary it should be the result of a conference with one or more physicians, in whose judgment confidence my be reposed. In order to save all embarrassment, and insure ourselves against any cloud of suspicion that might bring odium upon us, I concur in the opinion, that it is our imperative duty, whenever possible, to get the written consent of the mother, or husband, or guardian. For it is affirmed by the records in not a few instances, that suit for malpractice, or damaging reports have been the outcome of precipitate judgment, and a lack of regard for these precautions. Though it be justifiable, and so held by law, nevertheless by this inadvertent act, we inflict upon ourselves an irreparable injury, and place an ineffaceable blot upon our professional escutcheon. For the cause of the plaintiff, groundless though it be, still finds a champion. Therefore, let me not fail to forcibly impress your mind with the gravity of the responsibility that rests upon you, for in event any untoward sequelæ ensue, or as it has not infrequently happened, death be the sad termination, how much enhanced would your comfort be, that in the performance of this duty you had obeyed the dictates of law and conscience. Your position would then be rendered invulnerable and beyond a breath of reproach.

Of the various agencies resorted to by criminal abortionists in effecting this horrible purpose, it is manifest that they compass all known methods, from the most crude and barbaric to the most enlightened and scientific. They shall be mentioned only in a general way, and may be divided into two classes, *viz.:*

General or constitutional, and local or mechanical.

The general or constitutional seek to effect the expulsion through the constitution of the mother, and may be broadly classified, as, supposed or reputed abortifacients. These depend for their effectiveness upon an exciting uterine action, some of which have a direct, and some an indirect emmenagogic effect. The most prominent are emetics, purgatives, especially the drastic purgatives, such as croton oil, elaterium, gamboge, etc.

Diuretics.—Their administration usually proves fatal to the mother, owing to the necessarily large quantity required. Extract of cotton wood has been used in the Southern States, but is of uncertain action.

Carrot seed is a popular abortifacient in upper India.

Vegetable preparations: savine, rue, tansy, quinine, belladonna, ergot, etc.

Animal substances: cantharides, etc.

Each of these preparations and substances have certain authorities to claim for them the possession of potent abortifacient properties, while equally noted authorities will write avowing a negative opinion. Therefore, before reaching a conclusion, or determining a drug or substance to be effectual or ineffectual, we should not lose sight of the fact, that in women disposed to abort, the effect of the drug, conjointly with the mental impression, often proves sufficient to bring about an abortion; whereas, were we administering the same drug to a differently constituted, or less impressionable woman, it would be found devoid of any emmenagogic effect.

Of the local or mechanical causes, anything approaching a thorough enumeration, and description of the various means and methods, would indeed be prolix and out of order. Suffice it to say, that almost every conceivable device and horrible measure has been found in the employment of criminal abortionists in their attempts at the production of abortion. They direct their efforts toward the destruction of fetal life, or more properly speaking, the arresting of gestation.

This purpose has been accomplished by the destruction of the ovum, puncturing or separating the membrane, and a variety of other procedures, that light up an abnormal irritation and contraction of the uterus.

The fatality to the mother and terrible after-consequences, are attributable, in the greater number of cases, to haste, excitement, and septic conditions attending the work of an unskilled operator. It is here to be borne in mind that some days may elapse after puncturing the membrane, before the expulsion of the uterine contents; also a fatal inflammation may be set up without any actual injury to the womb.

At other times, death has resulted from the instrument being driven through the womb, and inflicting injury upon parts incredibly distant.

Horrible as it may appear, the human hand alone has been used with such extreme violence as to even drag away the intestines.

In instituting a plan of investigation I have adopted the one laid down by Tidy. He defines the duties of a medical expert, in cases of suspected abortion as stated below:

1st. Examination of the mother, if living.

Temperature.

As to the woman's predisposition to abort and the period at which abortion has commonly occurred.

General state of health (note existence of leucorrhea, excessive menstruations, syphilis, asthma, malignant disease, uterine disease, etc.)

Whether the woman be well or ill formed (note pelvic malformations, effects of tight lacing, etc.)

Whether or not there be signs of recent delivery, or of the expulsion of the uterine contents.

Whether any cause can be assigned to account for the abortion, (*e. g.*, violent coughing, blood-letting, straining at stool, violent exercise, undue excitement, septic poisoning, violence, administrations of medicine, etc.)

All injuries of the genital organs (consider whether the injuries might be self-inflicted.)

2d. Examination of the body of the mother, if dead.

The necessity for care not to mistake the effects of menstruation for those produced by abortion.

To avoid injuring the parts by the knife or otherwise during the autopsy.

To consider the possibility of injury being self-inflicted.

Note the existence of any marks of violence on the abdomen or other parts.

Condition of the genital organs, noting all inflammation, rents, tears, perforations, etc. (if the uterus be injured it should be preserved.)

Note also :

1. The condition of the passage (relaxed or otherwise.)
2. The condition of the os uteri (virginal or gaping, etc.)
3. Vaginal secretions, and if present, their character.
4. The general appearance of the breasts, presence of milk, etc.

Whether there be any signs of irritant poisoning in the stomach, or inflammation of the bladder, kidneys, rectum, etc. (The contents of the stomach, if necessary to be preserved.)

Whether the viscera generally indicates loss of blood during life.

3d. Examination of the products of conception.

Nature of the supposed product of conception.

Consider whether there is evidence of a diseased condition of the membranes, or of the placenta,—*e. g.*, structural degeneration. If a fetus be found, determine whether it was born alive, its probable age, and the cause of its death.

Determine whether, if there be wounds or other injuries, they were inflicted during life or after death.

4th. Examination of all drugs, instruments, etc.

If during life the examination be made, we must remember that the earlier it is done, the more certain are the signs, and if delayed much beyond a week or ten days, the evidence of recent delivery will at best be of an indefinite character. Confirmatory of this, an Indian surgeon of some experience writes that with some persons within twenty-four hours all signs of delivery disappear. This, of course, is very exceptional, as the lochia itself rarely ceases before a week or ten days.

### INFANTICIDE.

The thorough appreciation, and the ability to recognize infanticide, presupposes a knowledge replete with the many details surrounding the cause and effect of still birth, and the assigned meaning from a forensic point of view of the terms live-birth, and newly born. The term "still-birth," in its broad application, is corrupted, and would imply any child born dead, *i. e.* in common parlance, and often medically speaking. But when the law intervenes it has a definite and restricted meaning. To the medico-legal expert it conveys the idea of a child born dead, from natural causes, or inevitable causes arising from some justifiable procedure in the attempt at delivery.

Live-birth is stated by Bouvier in the following language, "The act of being wholly brought into the world. The conditions of live-birth are not satisfied when a part only of the body is born. The whole of the body must be brought into the world, and detached from that of the mother, and after this event the child must be alive. The circulating system must also be changed, and the child must have an independent circulation, but it is not necessary that there should have been a separation of the umbilical cord; that may still connect the child with its mother, and yet the killing of it will constitute murder."

We must satisfy our minds upon these several points, before judging the facts accordant with the legal conception of a live-birth.

It is here interesting to note the distinction between physiological life and legal life made by a distinguished judge in his charge to the jury in a trial for infanticide. "Medically," he said, this might be a live child, but legally it was not one, for in law, the birth of the child must be complete. As the result a prisoner who had murdered a child while being born by cutting off its head was acquitted.

I shall forego any amplification of this branch of my paper, further than the strictly medico-legal points, for by recourse to the abundant literature written in this connection, you have doubtless become aware, that it is teeming with statistical facts, and diffuse in its descriptions of recorded cases.

That it exists as a crime of measurably frequent occurrence, even in our own community, cannot be controverted, though seldom if ever does it take the shape of an indictable offence, owing to the lack of positive proof, though the assumptive is often very strong. I will here mention that on more than one occasion I have had my attention arrested and suspicion aroused by the conduct of mothers whose infants were insured by certain life insurance companies. The incentive for gain among a certain class, whose moral sensibilities are blunted, proves too much for them, and induces an apathy and extreme indifference, which deprives them of the ability to execute the injunctions enjoined by the attending physician. In all events, it is a singular and often to be observed fact, that there exists a noticeable want of motherly feeling and care among this element. In order to subject this to a crucial test, I will be glad if each member will feel it incumbent upon him to fathom the truth of my assertion, and if I be correct, I comprehend it from a moral standpoint to be the unswerving duty of this society to denounce it as a pernicious practice, and further to interest itself in preventing its continuance.

In an issue of the *British and Foreign Medical Review* an authority estimates the proportion of still-births to be one in eighteen, or one in twenty. This calculation is based upon a series of eight million births. A comparative ratio of still-births, among legitimate and illegitimate children, is supposed to be about one in twenty to the former, and about one in ten to the latter.

What constitutes a sufficient manifestation, or sign, to prove the existence of live-birth? In law we are not called upon to show that the life existed for any given length of time. A single second of life is all the law requires. The evidence, according to Tidy, may be of three kinds: First, the evidence of life furnished by an examination immediately after the birth. Second, the evidence of death, furnished by an examination immediately after the birth. Third, the evidence of live or of still-birth furnished by a post mortem examination.

*First:* The evidence of life furnished by an examination of the child immediately after its birth. These are muscular twitchings which *per se* would not be accepted as sufficient evidence of live-birth.

*Second:* Respiration—*i. e.*, breathing as evidenced by chest and other movements, is a true sign of life, but on the other hand, it does not follow because the child does not breathe, therefore it is not living.

*Third:* Crying after complete delivery is an indisputable sign of live-birth. It is not necessary, however, for the child to cry to prove live-birth, because it is possible it may be dumb. This is regarded as one of the strongest signs, as the act necessitates breathing.

*Fourth:* Pulsation in the cord. This is an undoubted sign. The pulsation being in the umbilical or hypogastric arteries and is due to the action of the child's heart.

*Fifth:* Beating of the heart. The beating of the child's heart is of all tests, the test of live-birth. Monsieur Bouchant records a case where the heart beat was audible for twenty-three hours after birth, although there were no signs of respiration during the time.

Evidence of live or still-birth furnished by a post mortem examination. We consider first the condition of the respiratory organs. The relationship of respiration to live-birth considered legally and physiologically is entirely different, for a child may breathe *in utero* (*vagitus uteranus*) whilst its head is in the vagina (*vagitus vaginatus*), or further, a child may breathe after the head only is external—*i. e.*, before the rest of the body is born, and still either, or all, of these facts would not constitute evidence of live-birth.

Our second inquiry is in regard to the shape of the chest and alteration in the position of the diaphragm. The diaphragm, before respiration, is arched and high up into the chest; after respiration, it is flattened and depressed.

The respective characters of lungs that have and have not breathed, may be concisely expressed by Tidy's tabular form denoting them:

| LUNGS WHICH HAVE NOT BREATHED. | LUNGS WHICH HAVE BREATHED. |
|---|---|
| 1st. Dark in color (black, blue, maroon, or purple), resembling liver. | 1st. Light in color (rose-pink, pale-pink, light-red, or crimson). |
| 2d. Air-vesicles not visible to the naked eye. | 2d. Air-vesicles distinctly visible to the naked eye, or to a lens of low power (say a two-inch or even a common reading glass). |
| 3d. Do not crepitate or crackle when squeezed or cut. | 3d. Crepitate or crackle freely. |
| 4th. Contain but little blood, therefore but little escapes on section | 4th. Contain a good deal of blood, which escapes freely on section. |
| 5th The blood present is not frothy, unless there be putrefaction. | 5th. This blood is freely mixed with air, and therefore appears frothy. |
| 6th. Sink in water, unless putrid, and often even then. | 6th. Float in water, or at all events, the parts which have been expanded, or have breathed, float. If fully expanded, they will even buoy up the heart. |
| 7th. Bubbles of gas arising from putrefaction may be squeezed out, and as they escape are usually noted to be of large size. | 7th. The air cannot be squeezed out. |

The extent of these changes depends upon the length of time the child breathed. Forderes or Schmit's test regarding the relative weight of respired or unrespired lungs is practically of no value. Plocquet's test, the ratio of weight of the lungs to weight of the body, is valueless. Speaking relatively to the hydrostatic test, which was first suggested by Ruggaet in 1862, it is based upon the specific gravity of the respired and unrespired lungs ; the specific gravity of the respired, when compared with water, is 1050, whilst that of the unrespired is 960.

My paper will not admit of a description of the mechanism of this test, which is fraught with difficulties, and involves a deep and close investigation in many particulars, and though it were not so at best, it could but prove the fact of respiration having taken place.

We are reminded that life and breathing are not convertible terms. It still remains but one of the proofs of live-birth, and by no means an exclusive one. Many reasons may be shown to evidence the fact that a child may be born alive and live for some hours without breathing. Amongst them, as pointed out by Dr. Hicks, in Guy's Hospital Reports of 1866, to exclude breathing, a spasm of the larynx and retraction of the tongue.

### THE LEGAL ASPECT IN GEORGIA.

I find by retrospective survey of the laws of Georgia that they are silent upon criminal abortion, prior to the act of 1876. That this crime is comparatively rare in the Southern States before the war cannot be doubted, yet I am at a complete loss to adduce a reason for its being so, and it would, in all probability, be a profitless and useless expenditure of mental energy to indulge in hypotheses offered in explanation, for unaltered would the fact remain. Yet I cannot refrain from reverting to certain historical facts that engrafted an indelible impress upon the character of that régime. These are the institutions peculiar to that period of Georgia's history, the common avocation of her people, that of agriculturists, and the fact of slavery being in operation, all of which, singly and conjointly, contributed their effect and reflected their influence upon the morals of society. Its existence, in a degree, is equally certain, and there is nothing to condone Georgia's failure to recognize it as a crime.

Not until twelve years after, when the aurora of another epoch had fully dawned upon her, when the crowning era of advancement marking her civilization was permanently established, and she finds the negroes and whites on a parity, legally, is she aroused to the necessity of enacting, in this direction, laws which purport to be the conservators of the public weal and the strong bulwark of morality. And what do we find to be the fruition of their puerile efforts? Naught more than inadequacy.

Let us scan them, and let us see if I have been guilty of perpetrating a dogma, unwarranted by facts, in making this broad assertion. For your edification upon the chief principles meriting your attention, I shall quote the several sections of the Code delineating the law, and if a full discussion tending to a correction of these laws be provoked, I shall have realized the *bonum desideratum* of my paper:

Section 4337(a). *Feticide, How Punished.*—The willful killing of an unborn child, so far developed as to be ordinarily called "quick," by any injury to the mother of such child, which would be murder if it resulted in the death of such mother, shall be guilty of a felony, and punishable by death or imprisonment for life, as the jury trying the case may recommend.

Section 4337(b). *Use of Medicine, Assault with Intent to Murder.*—Every person who shall administer to any woman pregnant with child any medicine, drug, or substance whatever, or shall use or employ any instrument or other means with intent thereby to destroy such child, unless the same shall have been necessary to preserve the life of such mother, or shall have been advised by two physicians to be necessary for such purpose, shall, in case the death of such child or mother be thereby produced, be declared guilty of an assault with intent to murder.

Section 4337(c). *Abortion, Punishment of.*—Any person who shall willfully administer to any pregnant woman any medicine, drug, or substance, or anything whatever, or shall employ any instrument or means whatever with intent thereby to produce the miscarriage or abortion of any such woman, unless the same shall have been necessary to preserve the life of such woman, or shall have been advised by two physicians to be necessary for that purpose, shall, upon conviction, be punished as prescribed in section 4310 of the Code.

Section 4310 provides for a fine not exceeding one thousand dollars, imprisonment not to exceed six months, to work on the chain-gang, on the public works, or on such other works as the county authorities may employ the chain-gang, not to exceed twelve months, and any one or more of these punishments may be ordered in the discretion of the judge.

THE MORAL ASPECT.

Criminal abortion and infanticide, the two most inhuman of crimes, directly opposed and in violation of the divine laws of God, of nature, and of man, appreciated doubtless by all in the abstract sense, yet I dare say that few of us are in possession of a concrete knowledge of these, of all evils the most horrible and abandoned. The latter, not nearly so common as the former, but of relatively frequent occurrence, its practice being confined, in a vast majority of instances, to the larger cities, notably London, Paris, New York, etc. Not until we view this subject in its entirety and resolve its direct and collateral effects to an analysis, are we awakened from our lethargy of indifference to a realization of the direful results entailed morally, socially, and physically. They are not to be exaggerated, and no language that could be used, be it never so potent, would accentuate with sufficient force the burning necessity of active co-operative service on the part of medicine and law in the employment of every means directed towards the suppression of these evils. 'Tis unquestionably culpable remissness on the part of the physician that connives at the existence of these crimes. His position should not be a neutral one. He should ever be ready, where there is tangible evidence of guilt, to put it to a test, and to suffer it to pass unnoticed is to criminate himself, if not in the sight of man, certainly in the sight of God.

I hold that one endowed with the inherent attributes of a true physician has an exalted duty to devolve upon him, which is not satisfied with the mere administering to the physical ills and the amelioration of suffering, but demands that his character and life shall bear the impress of an ennobled spirit. To morality he owes an inviolable allegiance, and should ever be actuated and governed by the purest impulses and God-inspired feeling of love, tending toward the promotion, among his associates and those to whom he is called upon to administer in the darkest moments of suffering and despair, of the highest and loftiest sense of Christian morality.

